# Supplementary material for: Size-dependent diffusion controls natural aging in aluminium alloys
Source: Nat Commun. 2019 Oct 18;10:4746. doi: 10.1038/s41467-019-12762-w (PMC6800430; doi:10.1038/s41467-019-12762-w)
Supplement: Supplementary file 1 — Supplementary Information [file 41467_2019_12762_MOESM1_ESM.pdf]

Size-dependent diffusion determines natural aging in aluminium alloys

Dumitraschkewitz et al.

Supplementary Information

Supplementary Note 1: Hardness evolution

Supplementary Fig. 1 illustrates the hardness evolution of the alloy 6016 during natural aging.

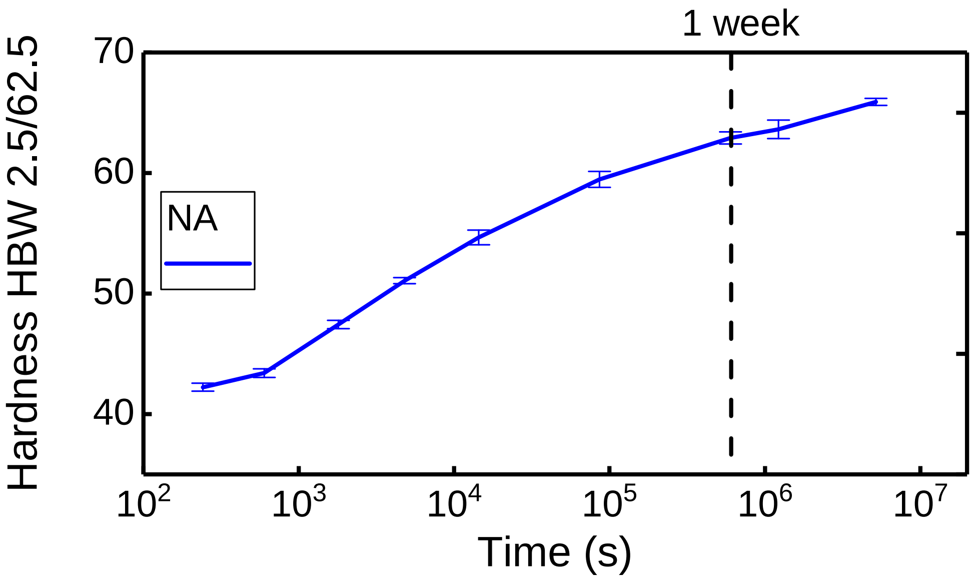

Supplementary Figure 1: Hardness evolution over natural aging time, after solution heat treatment and quenching. The dashed line illustrates one week of aging. Errorbars are calculated using the standard deviation of the measured values.

Supplementary Note 2: Pair correlation and radial distribution functions

The pair correlation function can be defined as [1]

$$g(R)_{AA} = \frac{\text{RDF}(R)_{AA}}{\rho 4\pi R^2 dR}, \tag{1}$$

where $\text{RDF}(R)_{AA}$ is the auto-correlation radial distribution function defined for atom probe data (discrete spatial points) for one atomic species. The equation enhanced for cross-correlation (AB), as also used in this study, is given in equation 2. Sometimes the left-hand side of equation 1 is called RDF [2], but we will use the terminologies of equations 1 and 2 through the manuscript. The denominator in equation 1 can itself be seen as the RDF of randomly distributed atom positions in an explicit form for an infinitly expanded medium. In atom probe tomography, the comparators for randomly distributed solute atoms are built with radom labeling, which means random sampling without replacement of the existing (x,y,z) coordinates for the given number of solutes. Equation 1 can thus be re-written to equation 3. For cross-correlation we define $\text{RDF}(R)_{AB,\text{rand}}$ as in equation 4, meaning that the positions of one species are fixed (B) while the other species (A) are randomly distributed on the remaining possible positions. Pair correlation values (equation 3) > 1 can already indicate clustering of solute atoms, but we use in this study the ratio of cumulative summed RDFs (equation 6). More than one random drawing is also applied to characterize the random distribution of solutes (equation 5). For the calculation of the measure $f_{AB}(r)$ the mean curve of the $h(r)_i$ random comparators are used (equation 6). The upper boundary for the transformed standard deviation (Std) of $h(r)_i$ is calculated via equation 7 and the lower one via equation 8.

$$\mathrm{RDF}\left(R\right)_{AB}=\sum_{k=1}^{n_A}\left(\sum_{\substack{l=1,\\ l\neq k \text{ if } A=B}}^{n_B}\left(\mathrm{Hist}\left(\left|\left|\vec{P}_{A,k}-\vec{P}_{B,l}\right|\right|_2\right)\right)\right) \tag{2}$$

$$g\left(R\right)_{AA}=\frac{\mathrm{RDF}\left(R\right)_{AA}}{\mathrm{RDF}\left(R\right)_{AA,\mathrm{rand}}} \tag{3}$$

$$\mathrm{RDF}\left(R\right)_{AB,\mathrm{rand}}=\sum_{k=1}^{n_A}\left(\sum_{\substack{l=1,\\ l\neq k \text{ if } A=B}}^{n_B}\left(\mathrm{Hist}\left(\left|\left|\vec{P}_{A_{\mathrm{rand}},k}-\vec{P}_{B,l}\right|\right|_2\right)\right)\right) \tag{4}$$

$$h(r)_i=\sum_{R=0}^{r}\left(\mathrm{RDF}\left(R\right)_{AB,\mathrm{rand},i}\right) \tag{5}$$

$$f_{AB}(r)=\frac{\sum_{R=0}^{r}\left(\mathrm{RDF}\left(R\right)_{AB}\right)}{\mathrm{Mean}\left(h\left(r\right)_i\right)},\ i=1...40 \tag{6}$$

$$\mathrm{err\_up}\left(f_{AB}(r)\right)=\frac{\sum_{R=0}^{r}\left(\mathrm{RDF}\left(R\right)_{AB}\right)}{\mathrm{Mean}\left(h(r)_i\right)-\mathrm{Std}\left(h(r)_i\right)} \tag{7}$$

$$\mathrm{err\_low}\left(f_{AB}(r)\right)=\frac{\sum_{R=0}^{r}\left(\mathrm{RDF}\left(R\right)_{AB}\right)}{\mathrm{Mean}\left(h(r)_i\right)+\mathrm{Std}\left(h(r)_i\right)} \tag{8}$$

Supplementary Note 3: Surface migration/surface relaxation and regions of interest

While values > 1 for the defined measure (equation 1) can indicate clustering, the measure is also sensitive to variations of densities for a specific species over a large scale. In general, APT reconstructions do not show uniform density, due to a faceting during the APT experiment. This leads to trajectory abberations resulting in ”poles”, which are regions of low density in the reconstruction and correspond to the respective crystal plane. In principle these density variations are considered by the use of a random labeling for the random comparator building, which resembles the density distribution in the reconstruction if the solute behaves such as the matrix species during the APT measurement.

For solutes with a higher evaporation field than the matrix, Si in Al, preferred retention can

occur depending on the experimental parameters, resulting in surface migration/surface relaxation [3, 4, 5] during the APT experiment. This means that Si may be prefentially retained (in a stochastical process) on the sample surface and thus have the possibility to move in the high-field environment accross the surface to a certain direction (surface migration) [3], or athermally relax into a plane position of a certain direction (surface relaxation) [5] – leading to Si which appears at another position in the reconstruction than originally in the sample. It is generally argued that Si moves towards high-field directions [3] of the crystal or is relaxed into a higher-neighbor-count position [5]. The amount of Si surface migration is especially large if the (111) pole [4] (Supplementary Fig. 4 and Supplementary Fig. 8) or the zone line (111)-(022) (Supplementary Fig. 7) is visible on the detector hitmap. In general this issue is encountered by avoiding influenced regions and choosing subsets of the data, i.e. regions of interest.

Supplementary Fig. 2 illustrates low Si-Si ratios for the whole datasets "RAW" with the signals decreasing over the natural aging times for both samples (nano_aged_01 and nano_aged_02). In general, the Si-Si signal of the "nano_aged_02" specimen is higher than the "nano_aged_01" specimen for the whole dataset and does not decay to unity within 5 nm. For "nano_aged_02" the zone line (111)-(022) and the (111) pole were obtained (Supplementary Fig. 4), and increased Si hits at the zone line (111)-(022) and the (111) pole were seen (Si surface migration). A region of interest was thus chosen and the data near the (111) pole were neglected from evaluation (darkened regions in Supplementary Fig. 4). The Si-Si signal then generally dropped and for $r > 2$ nm the signal now became close to unity (Supplementary Fig. 2 "ROI"; data from main text Fig. 2), but the sequence of the signals remained (Si-$Si_{30\ min} >$ Si-$Si_{130\ min}$). Although the region of interest neglect the (111) pole, there is still a part of the zone line present and also the Si-depleted regions next to the (111) pole region cause a non-random signal. For "nano_aged_01" no (111) pole or (111)-(022) zone line were visible and also Si-Si values of unity were obtained for $r > 2$ (Supplementary Fig. 2 "RAW"). Choosing a region of interest

around the (002) pole (Supplementary Fig. 3) does not alternate the Si-Si signal significantly, and the sequence of the signals are again retained and highest for low NA times, with a trend of decreasing signal with increasing NA time. Inspecting the chosen region of interest in detail for ”nano_aged_01” (Supplementary Fig. 3) for Si reveals that slightly depleted regions are present along the zone lines of the (002) poles (cross shaped). If we compare the Si hitmaps of ”nano_aged_01” for 10 min of NA and 3 weeks of NA in Supplementary Fig. 5 we obtain larger Si-depleted regions at the zone lines for 10 min NA, which cause an increased non-random signal. If we take a smaller region of interest for ”nano_aged_01” at 10 min of NA at a position that appears more unifom at the detector hitmap (Supplementary Fig. 6) and re-evaluate the spatial distribution, we obtain again reduced values close to unity, now for almost all r values.

We attribute the small Si-Si signals in Supplementary Fig. 2 and maint text Fig. 2 to density variations in Si over a large scale caused by Si surface migration. Regarding the fact that the signals decrease for increasing NA time, we assume that the traveling distance of Si at the surface stays constant during the APT measurement, while the overall sample surface area increases. This overall decreases the signal from migrating Si atoms with increasing sample radius (Supplementary Fig. 5) and is not caused by NA. The effect only correlates with the increased NA time because in the current *in-situ* aging experiments a higher NA time implies a larger radius.

The chosen regions of interest for all specimen are given in Supplementary Figs. 3, 4, Fig. 7, 8 and 9. In general, it should be noted that choosing a perfect region of interest may be difficult if two species (Si, Mg) with different field-evaporation characteristics (Si has a higher and Mg has a lower evaporation field than Al) need to be investigated. The changes from choosing the whole dataset to choosing the regions of interest for the bulk aging experiments are given in Supplementary Fig. 10.

Supplementary Table 1: Overview of experiments and samples.

Sample name	Time bulk RT[1]	Prep. T[2]	Nano aging time at RT[3]	Mg[4][%]	Si[5][%]	Size[6]
nano_aged_01	0	-40° C	10’	0.27	0.97	5.2
			30’	0.27	0.96	5.6
			60’	0.27	0.95	5.6
			180’	0.26	0.94	6.3
			360’	0.26	0.94	6.3
			23h	0.26	0.93	7.6
			1 week	0.26	0.95	8.9
			3 weeks	0.25	0.92	11.5
nano_aged_02	0	-40° C	30’	0.27	1.05	9
			130’	0.26	1.00	6.9
bulk_aged_01	9’	RT	3 weeks	0.36	1.02	21.4
			6 weeks	0.35	1.01	10.2
bulk_aged_02	1 week	RT	2 weeks	0.40	1.20	6.3
bulk_aged_03	1 week	RT	1 day	0.38	0.97	2.8

[1] Time at room temperature after quenching at bulk dimensions (rods $0.7\times0.7\times20$ mm).
[2] Ambient temperature of the sample preparation location.
[3] Time during natural aging in the nano (APT specimen) dimensions.
[4] Mg, molar percent of the used reconstruction, no un-ranged ions counted.
[5] Si, molar percent of the used reconstruction, no un-ranged ions counted.
[6] Number of atoms, in millions, for the used .pos file (whole dataset).

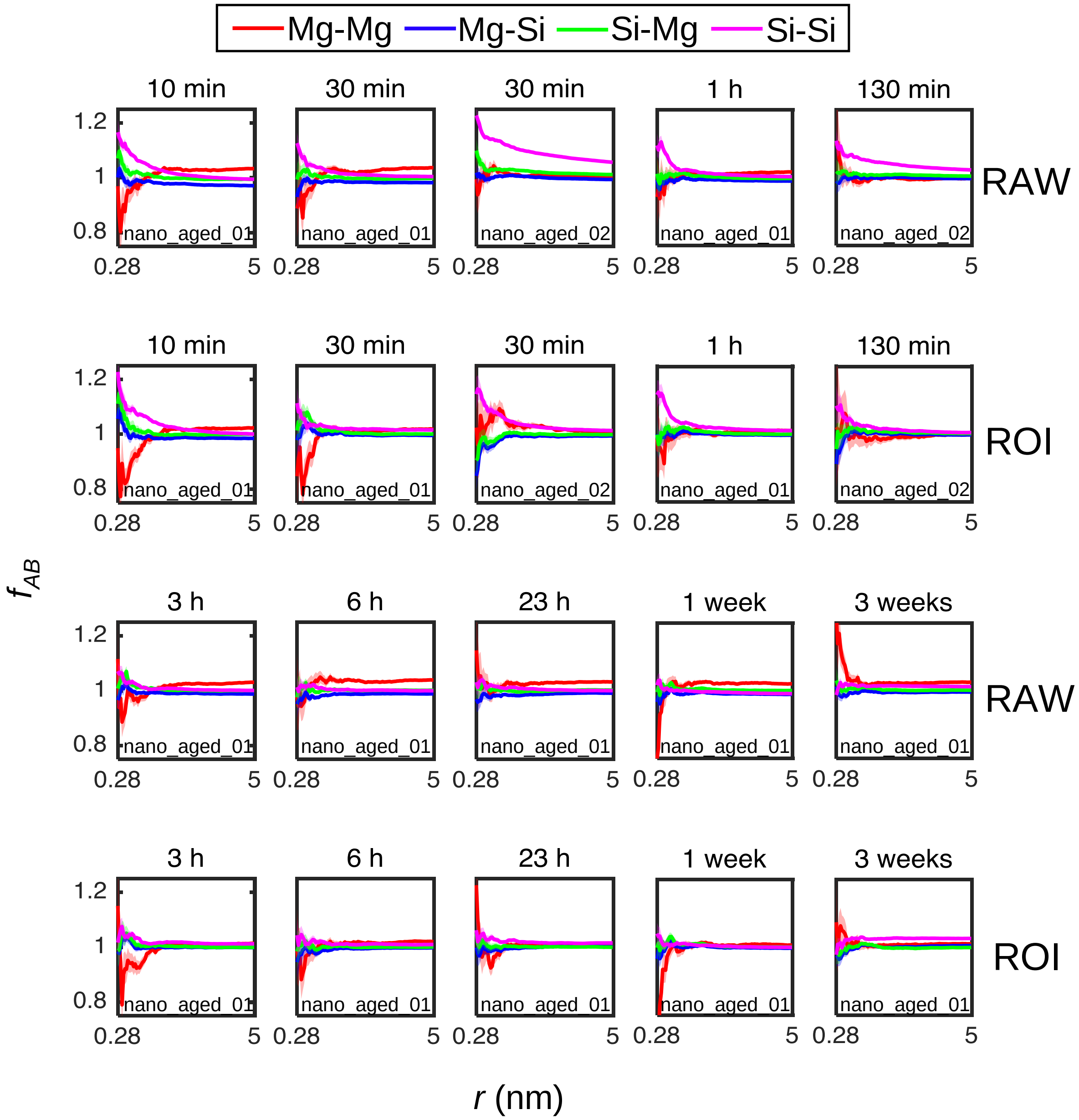

Supplementary Figure 2: Influence of choosing regions of interest (ROI) on the evaluation measure (equation 1). *In-situ* natural aging after solution heat treatment and preparation of samples at -40° C (nano aging). Analysis of the spatial positions of the solute atoms for the given interactions (Mg-Mg, Si-Si, Si-Mg and Mg-Si) of "nano_aged" samples, see Supplementary Table 1. Values $f_{AB} > 1$ indicate clustering. The results labeled "RAW", correspond to the whole dataset, whereas the results labeled as "ROI" (same data as main text Fig. 2) correspond to the regions of interest. Errorbar boundaries are calculated according to equation 7 and 8.

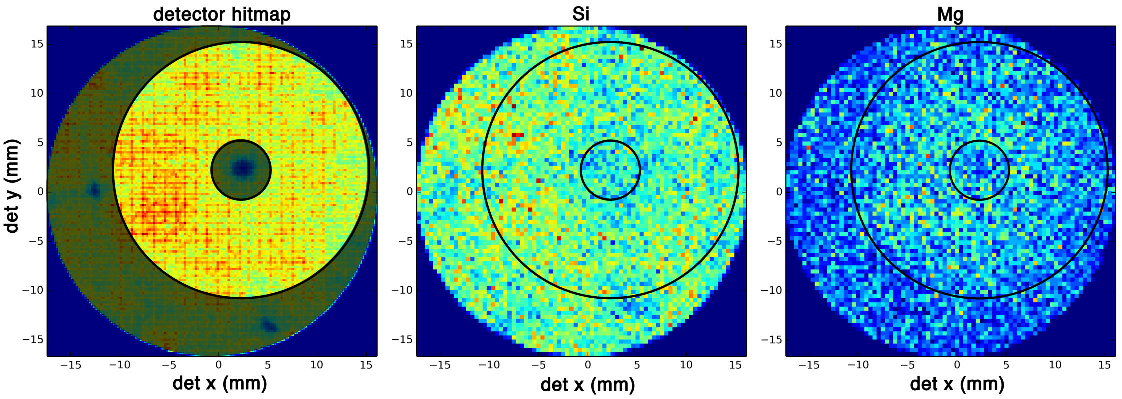

Supplementary Figure 3: ”nano_aged_01” detector hitmaps for all atoms, Si atoms, and Mg atoms, respectively. Regions of interest: Neglected data are darkened in the detector hitmap and the borderline used is drawn. The used borderlines are also drawn in the Si and Mg detector hitmaps. The region of interest is centered around the (002) pole.

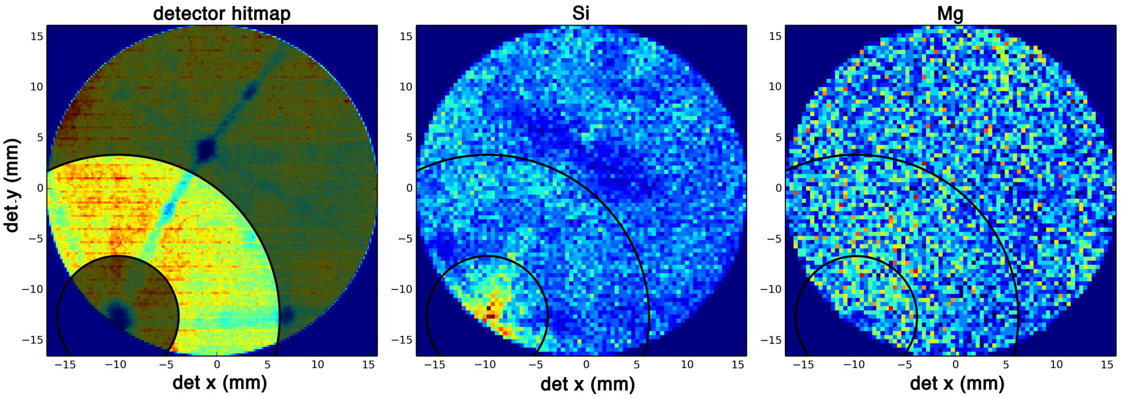

Supplementary Figure 4: ”nano_aged_02” detector hitmaps for all atoms, Si atoms, and Mg atoms, respectively. Regions of interest: Neglected data are darkened in the detector hitmap and the borderline used is drawn. The used borderlines are also drawn in the Si and Mg detector hitmaps. The region of interest is centered around the (111) pole.

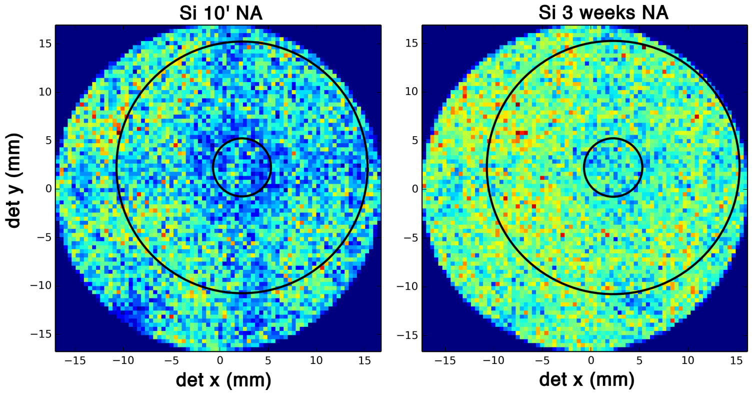

Supplementary Figure 5: ”nano_aged_01” detector hitmaps for Si atoms for 10 minutes and 3 weeks NA. The used borderlines for the regions of interest are drawn. The region of interest is centered around the (002) pole. For 10 minutes of NA a larger depleted region at the positions of the zone lines is obtained.

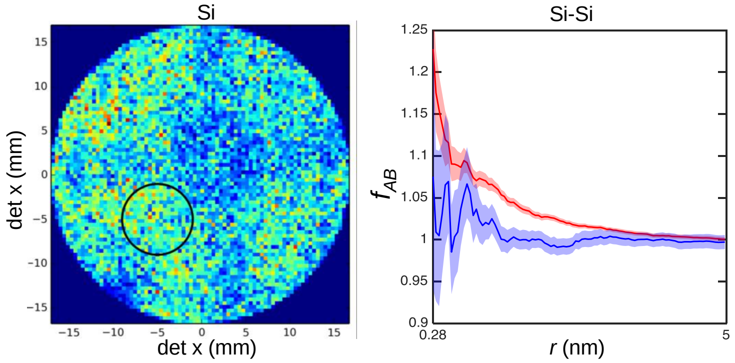

Supplementary Figure 6: ”nano_aged_01” (10 minutes of NA) detector hitmap for Si atoms and a differently chosen ROI. The used borderlines for ROIs are drawn. The ROI is placed on a position of uniform hit density for Si on the detector hitmap, and the chosen ROI has a diameter of approximately 18 nm in the reconstruction. The spatial distribution for Si-Si is analyzed for the ROI and compared to the analysis of the previously used ROI in Supplementary Fig. 2. Errorbar boundaries are calculated according to equation 7 and 8.

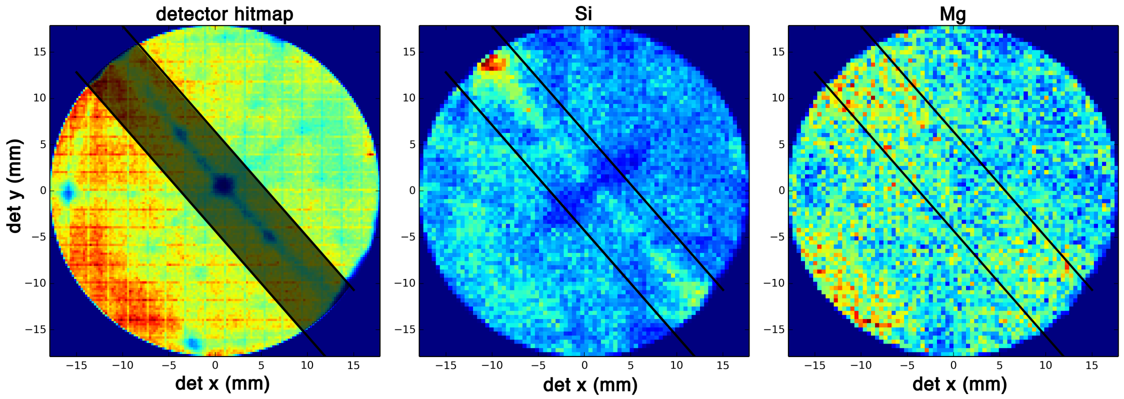

Supplementary Figure 7: ”bulk_aged_01” detector hitmaps for all atoms, Si atoms, and Mg atoms, respectively. Regions of interest: Neglected data are darkened in the detector hitmap and the borderline used is drawn. The used borderlines are also drawn in the Si and Mg detector hitmaps. The neglected data is near the (111)-(022) zone line, the region of interest is the remaining area.

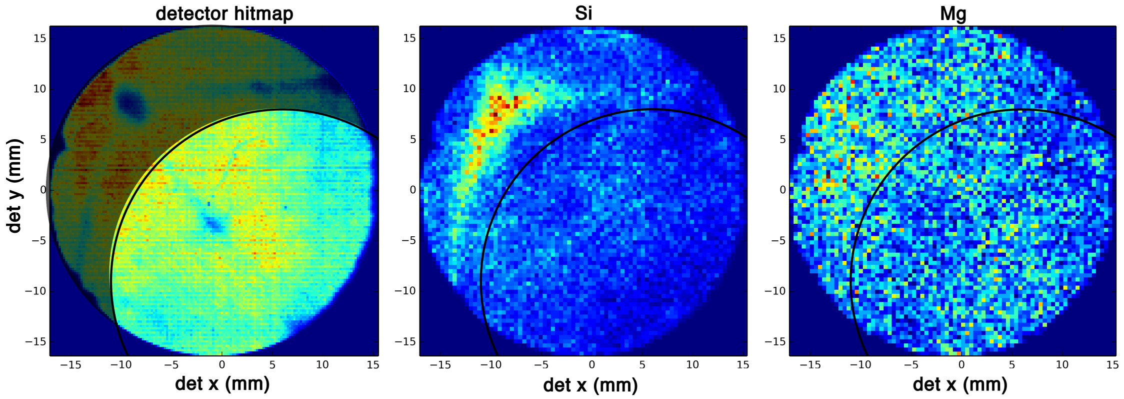

Supplementary Figure 8: ”bulk_aged_02” detector hitmaps for all atoms, Si atoms, and Mg atoms, respectively. Regions of interest: Neglected data are darkened in the detector hitmap and the borderline used is drawn. The used borderlines are also drawn in the Si and Mg detector hitmaps. The (002) pole is seen at the edge of the detector hitmap in the fourth quadrant.

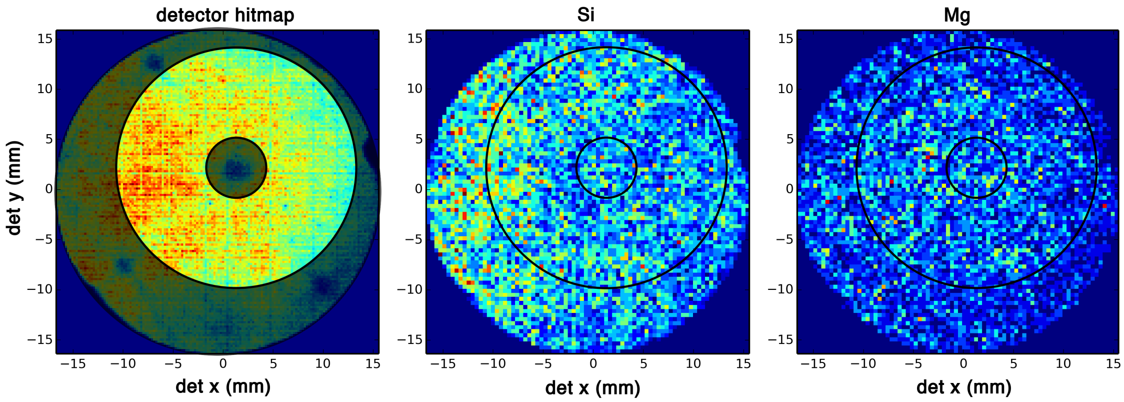

Supplementary Figure 9: ”bulk_aged_03” detector hitmaps for all atoms, Si atoms, and Mg atoms, respectively. Regions of interest: Neglected data are darkened in the detector hitmap and the borderline used is drawn. The used borderlines are also drawn in the Si and Mg detector hitmaps. The region of interest is centered around the (002) pole.

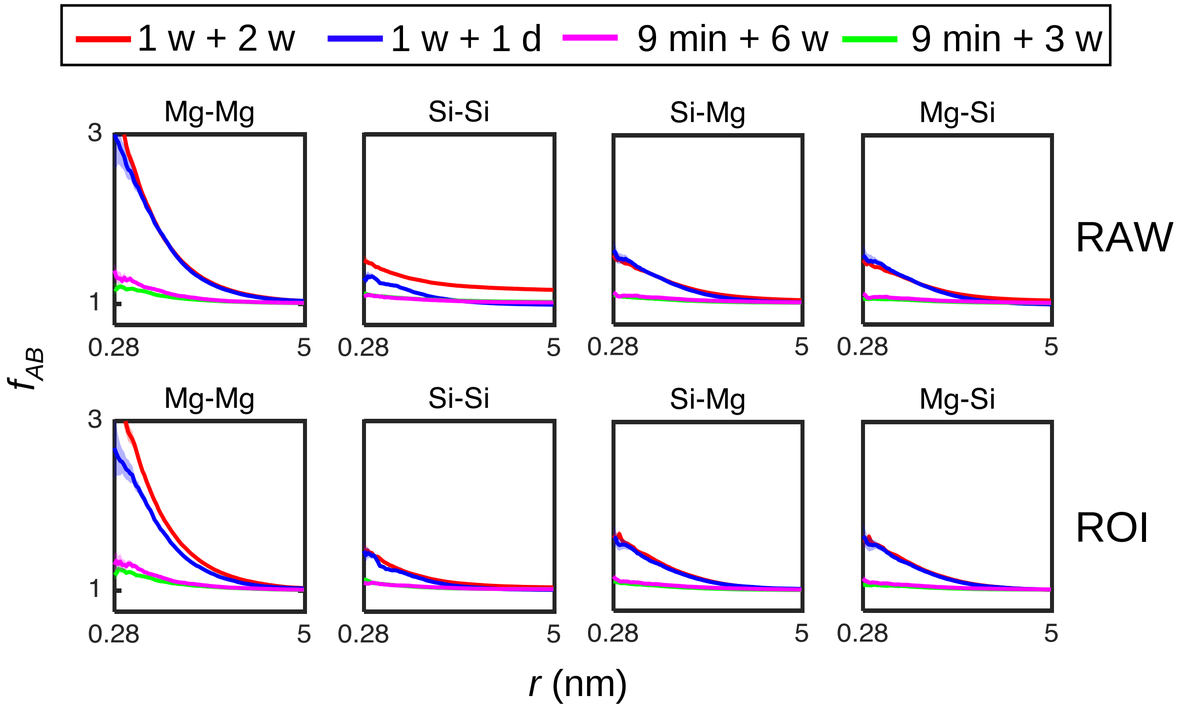

Supplementary Figure 10: Bulk-aging after solution heat treatment and further nano aging. Spatial analysis is calculated for the solute interactions Mg-Mg, Si-Si, Si-Mg and Mg-Si. Values $f_{AB} > 1$ indicate clustering. The investigated heat treatment states are labeled as ”bulk aging time + nano aging time”, e.g. ”1 w + 2 w” for one week bulk aging with additional two weeks nano aging. Shown are the results for the whole dataset ”RAW”, and the results for the region of interests ”ROI”. The ”1 w + 1 d” heat treatment (blue) shows curves corresponding to ”1 w + 2 w” (red), further the ”9 min + 3 w” (green) and ”9 min + 6 w” (purple) heat treatments show almost no difference. Note that ”9 min + 6 weeks” is the further nano aged ”9 min + 3 w” sample. Errorbar boundaries are calculated according to equation 7 and 8.

Supplementary Table 2: Cluster-search parameters for the "create cluster analysis" method using IVAS 3.6.12, and resulting number densities. References from which the used cluster-search parameters were obtained are also listed.

d_{max}	Order	N_{min}	L	d_{erode}	Nr. density $\times 10^{22}$ $1/m^3$	Ref.	ref. Nr. density$\times 10^{22}$ $1/m^3$
0.700	1	10	0.700	0.00	58	[7, 8, 9]	122[1]
0.750	1	10	0.750	0.00	108	[10]	130[2]

[1] 1 week of NA, LAR3DAP ($\sim$ detection efficiency as reflectron-fitted LEAP), 0.51% Mg, 0.94% Si atomic, maximum separation cluster search [11], (d_{max}, N_{min}), Data in Brief Table 6.

[2] 300 h (12 days) of NA , LEAP 3000 HR [12], 0.62% Mg, 0.93% Si mass, maximum separation cluster search [13], (d_{max}, N_{min}), datapoint from Fig. 6.

Supplementary Note 4: Cluster search

For comparison to literature data, a cluster search with the program IVAS 3.6.12 ("create cluster analysis") was carried out for the sample "bulk_aged_02". The used cluster search parameters and resulting number densities are given in Supplementary Table 2. The number density is not a very robust measure and can strongly depend on the input parameters. However, our measurements correspond to the values of the literature for 1 week of NA, despite the fact that several limitations need to be considered. The maximum seperation cluster search may for example depend on the software version used for analysis [6] and different compositions are used in different literature studies. It is visually obtained that the parameters from Ref. [7, 8, 9] for this dataset lead to an underestimation of the amount of clusters, possibly due to the difference in solute content.

Supplementary Note 5: Non-equilibrium vacancy evolution

For completeness Supplementary Fig. 11a shows simulations of the vacancy evolution for the used alloy with vacancy trapping for Mg and Si in comparison to the results of main text Fig. 3. In addition, Supplementary Fig. 11b shows the calculated impact of an increased dislocation

density for pure Al.

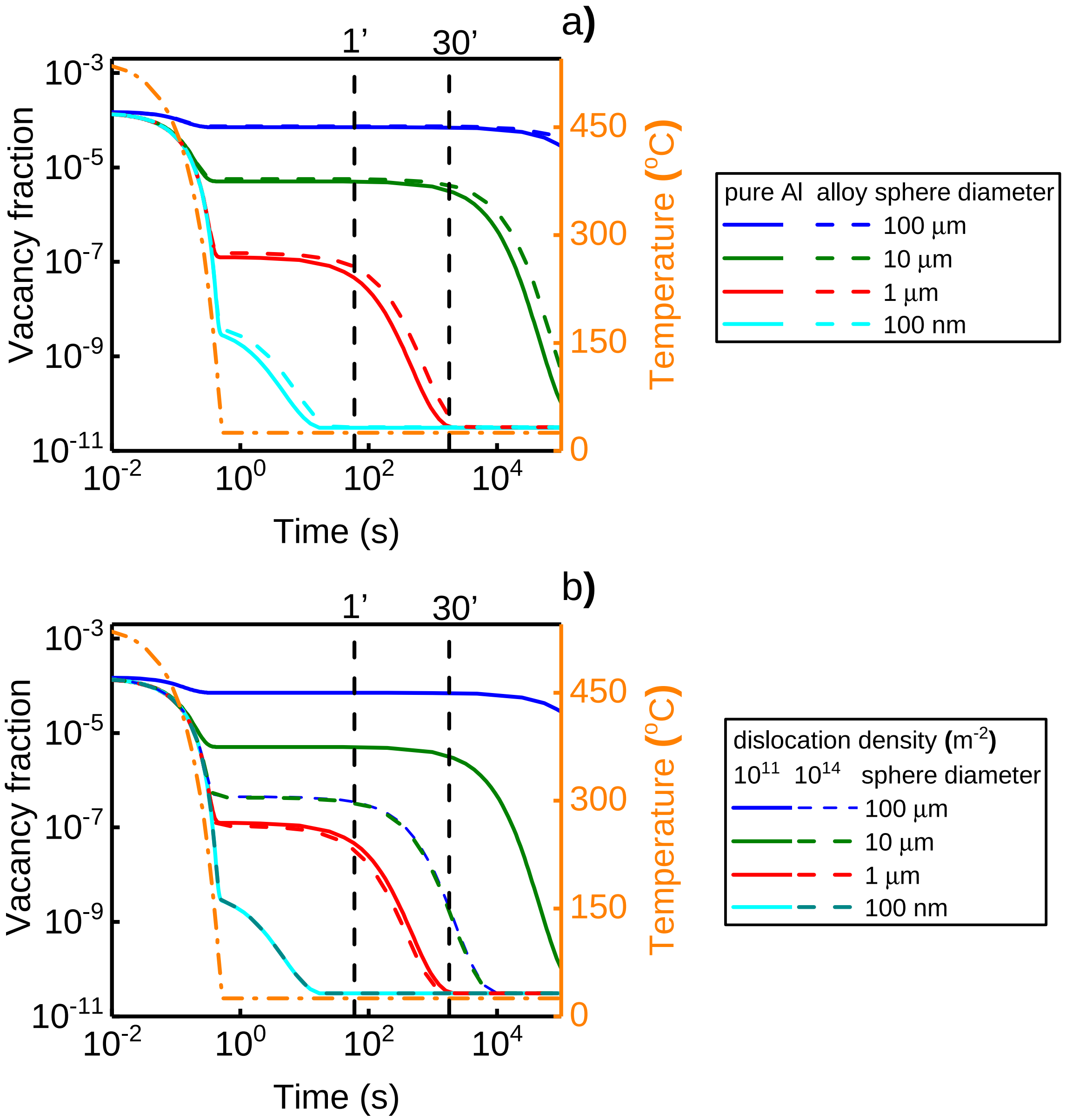

Supplementary Figure 11: a) Simulation of the non-equilibrium vacancy evolution. Calculated non-equilibrium vacancy fraction over time and temperature upon quenching and natural aging for pure Al and the used alloy (dashed lines) with solutes as trapping sites. Vacancy binding energies of 5000, 1000, and 0 J mol^{-1} were used for Si, Mg and Cu respectively [14]. b) Simulation of of the non-equilibrium vacancy evolution for pure Al with $\times 10^3$ increased dislocation density. A limiting effective vacancy-to-sink distance, lying between the sphere diameter of 1 μm and 10 μm, is introduced with increasing dislocation density. Additional lines for 1 minutes and 30 minutes are added as visual guidelines.

Supplementary References

[1] de Geuser, F., Lefebvre, W. & Blavette, D. 3D atom probe study of solute atoms clustering during natural ageing and pre-ageing of an Al-Mg-Si alloy. *Philosophical Magazine Letters* **86**, 227–234 (2006).

[2] Haley, D., Petersen, T., Barton, G. & Ringer, S. P. Influence of field evaporation on radial distribution functions in atom probe tomography. *Philosophical Magazine* **89**, 925–943 (2009).

[3] Gault, B., Danoix, F., Hoummada, K., Mangelinck, D. & Leitner, H. Impact of directional walk on atom probe microanalysis. *Ultramicroscopy* **113**, 182–191 (2012).

[4] Pogatscher, S., Gerstl, S., Löffler, J. F. & Uggowitzer, P. J. Atom probe tomography investigations of modified early stage clustering in Si-containing aluminum alloys. *Acta Physica Polonica A* **128**, 643–647 (2015).

[5] Oberdorfer, C. *et al.* Influence of surface relaxation on solute atoms positioning within atom probe tomography reconstructions. *Materials Characterization* **146**, 324–335 (2018).

[6] Cao, L., Rometsch, P. A. & Couper, M. J. Clustering behaviour in an Al–Mg–Si–Cu alloy during natural ageing and subsequent under-ageing. *Materials Science and Engineering: A* **559**, 257–261 (2013).

[7] Zandbergen, M. W., Xu, Q., Cerezo, A. & Smith, G. Study of precipitation in Al–Mg–Si alloys by atom probe tomography I. Microstructural changes as a function of ageing temperature. *Acta Materialia* **101**, 136–148 (2015).

[8] Zandbergen, M. W., Cerezo, A. & Smith, G. Study of precipitation in Al–Mg–Si alloys by atom probe tomography II. Influence of Cu additions. *Acta Materialia* **101**, 149–158 (2015).

[9] Zandbergen, M. W., Xu, Q., Cerezo, A. & Smith, G. Data analysis and other considerations concerning the study of precipitation in Al–Mg–Si alloys by atom probe tomography. *Data in Brief* **5**, 626–641 (2015).

[10] Aruga, Y., Kozuka, M., Takaki, Y. & Sato, T. Formation and reversion of clusters during natural aging and subsequent artificial aging in an Al–Mg–Si alloy. *Materials Science and Engineering: A* **631**, 86–96 (2015).

[11] Vaumousse, D., Cerezo, A. & Warren, P. J. A procedure for quantification of precipitate microstructures from three-dimensional atom probe data. *Ultramicroscopy* **95**, 215–221 (2003).

[12] Aruga, Y., Kozuka, M., Takaki, Y. & Sato, T. Evaluation of solute clusters associated with bake-hardening response in isothermal aged Al-Mg-Si alloys using a three-dimensional atom probe. *Metallurgical and Materials Transactions A* **45**, 5906–5913 (2014).

[13] Hyde, J. M. & English, C. A. An analysis of the structure of irradiation induced Cu-enriched clusters in low and high nickel welds. *MRS Proceedings* **650**, R6.6 (2000).

[14] Simonovic, D. & Sluiter, M. H. F. Impurity diffusion activation energies in Al from first principles. *Physical Review B* **79**, 054304 (2009).
